# Patient-reported outcome measures for systemic lupus erythematosus clinical trials: a review of content validity, face validity and psychometric performance

**DOI:** 10.1186/s12955-014-0116-1

**Published:** 2014-07-22

**Authors:** Laura Holloway, Louise Humphrey, Louise Heron, Claire Pilling, Helen Kitchen, Lise Højbjerre, Martin Strandberg-Larsen, Brian Bekker Hansen

**Affiliations:** 1 Adelphi Values, Adelphi Mill, Bollington, Macclesfield SK10 5JB, Cheshire, UK; 2 Abacus International, Manchester One, Manchester M1 3LD, UK; 3 The University of Manchester, Oxford Road, Manchester M13 9PL, UK; 4 Novo Nordisk A/S, Novo Allé 1, Bagsvaerd, 2880, Denmark

**Keywords:** Systemic lupus erythematosus, Patient-reported outcomes, Conceptual model, Fatigue, Health-related quality of life

## Abstract

**Background:**

Despite overall progress in treatment of autoimmune diseases, patients with systemic lupus erythematosus (SLE) experience many inflammatory symptoms representing an unmet medical need. This study aimed to create a conceptual model of the humanistic and economic burden of SLE, and review the patient-reported outcomes (PROs) used to measure such concepts in SLE clinical trials.

**Methods:**

A conceptual model for SLE was developed from structured review of published articles from 2007 to August 2013 identified from literature databases (MEDLINE, EMBASE, PsycINFO, EconLit) plus other sources (PROLabels, FDA/EMA websites, Clinicaltrials.gov). PROs targeting key symptoms/impacts were identified from the literature. They were reviewed in the context of available guidance and assessed for face and content validity and psychometric properties to determine appropriateness for use in SLE trials.

**Results:**

The conceptual model identified fatigue, pain, cognition, daily activities, emotional well-being, physical/social functioning and work productivity as key SLE concepts. Of the 68 articles reviewed, 38 reported PRO data. From these and the other sources, 15 PROs were selected for review, including SLE-specific health-related quality of life (HRQoL) measures (n = 5), work productivity (n = 1), and generic measures of fatigue (n = 3), pain (n = 2), depression (n = 2) and HRQoL (n = 2). The Functional Assessment of Chronic Illness Therapy - Fatigue Scale (FACIT-Fatigue), Brief Pain Inventory (BPI-SF) and LupusQoL demonstrated the strongest face validity, conceptual coverage and psychometric properties measuring key concepts in the conceptual model. All PROs reviewed, except for three Lupus-specific measures, lacked qualitative SLE patient involvement during development. The Hospital Anxiety and Depression Scale (HADS), Short Form [36 item] Health Survey version 2 (SF-36v2), EuroQoL 5-dimensions (EQ-5D-3L and EQ-5D-5L) and Work Productivity and Activity Impairment Questionnaire: Lupus (WPAI:Lupus) showed suitability for SLE economic models.

**Conclusions:**

Based on the identification of key symptoms and impacts of SLE using a scientifically sound conceptual model, we conclude that SLE is a condition associated with high unmet need and considerable burden to patients. This review highlights the availability and need for disease-specific and generic patient-reported measures of relevant domains of disease signs and symptoms, HRQoL and work productivity, providing useful insight for SLE clinical trial design.

## Background

Systemic lupus erythematosus (SLE) is a heterogeneous, inflammatory, multisystem autoimmune disease. Estimates of overall prevalence rates (per 100,000) vary worldwide, ranging from 4–45 in Asia-Pacific countries [1], to 52–150 in the USA [2],[3]. The prevalence of SLE is greater in non-white racial groups [2] and the disease affects women more frequently than men [4]. SLE is associated with a substantial economic burden, with direct costs per patient-year ranging from $3,735 to $14,410 [5].

There is a large variation in SLE associated symptoms and the condition is often complicated by flares (exacerbations) of varying severity and subsequent remissions [6]. A recent international consensus working group defined a flare as “a measurable increase in disease activity in one or more organ systems involving new or worse clinical signs and symptoms and/or laboratory measurements” [7].

Patients with active SLE experience musculoskeletal and mucocutaneous manifestations, including joint pain and swelling, skin rash and fatigue [8]. Frequently affected joints include the fingers, hands, wrists and knees, with some patients developing secondary osteoarthritis [8]. In addition to joint inflammation, internal organ involvement can be of greater concern when considering patients’ prognosis, with SLE often affecting the heart, lungs, blood vessels, liver, kidneys and nervous system [8],[9]. Overall these symptoms and manifestations can contribute to a substantially reduced health-related quality of life (HRQoL) [10].

It is not possible to assess many of the symptoms and treatment effects associated with SLE using objective clinical measures alone. Regulatory bodies and healthcare decision makers recognise the importance of also capturing the patient perspective in clinical trials by using validated and reliable patient-reported outcome (PRO) measures [11]–[13]. The U.S. Food and Drug Administration (FDA) and the European Medicines Agency (EMA) have released guidance which highlights the importance of measuring fatigue in clinical trials in SLE [14],[15] (although the FDA does not necessarily consider existing measures of fatigue to be adequate), and the EMA also strongly recommends the consideration of the impact of SLE on patients’ HRQoL [15]. In 1999 an Outcome Measures in Rheumatology group (OMERACT) performed a review of outcome measures that have been used in SLE trials and made recommendations regarding the most important domains to assess, and the most appropriate instruments to do so [16],[17]. However, as it has been a number of years since that review, there is value in a more up to date review of the literature which identifies relevant concepts for measurement and the adequacy of existing measures to assess those concepts. In particular, no disease-specific measures of health status or disability were identified at that time. An understanding of the symptoms and their impact on patients’ daily lives, the economic burden and the PRO measures available, will help researchers and clinicians evaluate the efficacy and impact of interventions.

The objectives of this research were twofold. The objective of the first stage of this research was to review and describe the symptoms and impact concepts of SLE from the patient’s perspective by means of a conceptual model. This included an overview of the economic burden of SLE. Based on development of the conceptual model, the objective was then to review existing PRO measures for suitability in clinical trials of SLE, in terms of their content validity, face validity and psychometric properties.

## Methods

A structured literature review was conducted to establish the humanistic and economic burden with respect to the key symptoms and their impact, and the PROs available to measure these. Methods were in line with recognised international guidelines for the conduct and reporting of literature reviews [18],[19]. The findings from the literature review informed the development of a conceptual model that was used to assess whether the selected PRO measures target the key symptoms and impacts of SLE in accordance with the EMA and FDA PRO guidance [11],[12].

### Data sources and searches

Literature searches were conducted in MEDLINE, EMBASE, PsycINFO and EconLit, limited to humans, English language and articles published between 2007 and 2013. The search was conducted on 1st August 2013.

### Inclusion and exclusion criteria

Journal articles (excluding conference abstracts, dissertations and book chapters) containing the keywords in the title and/or abstract were included. Articles with an SLE-related clinical search term and at least one of the humanistic or economic search terms were selected.

Search terms included **SLE clinical terms** (systemic lupus erythematosus, SLE, lupus nephritis, LN) plus **outcomes research terms** (health-related quality of life, quality of life, patient burden, patient impact, burden of illness, symptom, activities of daily living, patient reported outcome, patient related outcome, PRO, questionnaire, fatigue, physical function, emotion, mood) or **economic terms** (cost OR cost utility, cost of illness, healthcare cost, economic burden, economic impact, resource use, hospitalisation, productivity, expenditure, direct costs, indirect costs, economic, cost minimisation, burden of illness, cost effectiveness).

### Screening process and data extraction

Two researchers screened and checked all the abstracts for eligibility and suitability in line with the Centre for Reviews and Dissemination guidance [19]. Articles selected for review were based on consensus opinion. Any disagreement in the selection of articles was resolved by the lead researcher who performed a final, independent review of titles and abstracts. The selected articles were categorised into qualitative studies reporting burden from the patient perspective and articles reporting humanistic and economic burden data. Literature documenting quantitative methods was also reviewed to further understand the symptoms of SLE, the impact on patients’ HRQoL and to support the conceptual model. Study characteristics from the selected articles were extracted.

### Development of the conceptual model of systemic lupus erythematosus

A conceptual model of the humanistic and economic burden of SLE was developed on the basis of the review of qualitative studies [20]. A conceptual model can help identify themes, describe the patient burden concepts and their interrelationships and provide the rationale for PRO measures of interest [11],[21]. For the purposes of this study, a conceptual model for SLE provided the theoretical basis for the review and evaluation of the content validity of the selected PRO measures. The model was then compared with an existing SLE conceptual model developed by Gallop *et al.*[22] in 2012.

### Review of the patient-reported outcome measures for systemic lupus erythematosus

A subset of the PRO measures identified in the literature search were selected for in-depth review. Selection of instruments for in-depth review was based on likelihood of meeting the FDA guidance and relevance of conceptual coverage, based on review of the abstract and initial review of the PRO. Searches of PROQOLID, PROLabels, FDA/EMA websites, Clinicaltrials.gov and reimbursement agencies’ websites (e.g. National Institute of Health and Care Excellence (NICE, UK) and the Institute for Quality and Efficiency in Healthcare (IQWIG, Germany)) were conducted when either no relevant PRO that measured a key concept could be identified from the literature, or when expert opinion suggested the use of alternative PROs. Other PRO measures were only included in the review if the PRO was developed or validated in patients with SLE, had been previously used in a SLE population and/or if there was evidence of normative data available for comparison. Articles in the literature search that reported PRO data were categorised according to the type of PRO measure included in each study. The categories were largely based on the key concepts identified in the conceptual model.

Each PRO was reviewed for appropriateness for clinical trials in patients with SLE in terms of content validity, face validity and psychometric properties, in line with regulatory guidance for the evaluation of PRO measures [11],[12]. Content validity was assessed by the coverage level of the concepts within the conceptual model [11],[12]. The face validity of each PRO measure was determined by the acceptability and appropriateness of item wording, recall period and response options to patients with SLE. The level of qualitative research involved in developing each PRO was also assessed, acknowledging the value of input from the target patient population. The psychometric properties (validity, reliability and ability to detect change) of each PRO for an SLE population were also assessed in line with FDA regulatory guidance for the development and validation of PROs [11],[23].

## Results

### Findings from the literature search

The literature search identified a total of 1,754 publications. Of these, 687 articles met the inclusion criteria and the full articles were retrieved. A review of these identified 30 articles containing data on the economic burden of SLE. These were used to describe the economic burden in the conceptual model. The remaining 38 articles were related to patient-reported symptoms, impacts and burden in patients with SLE.

### Conceptual model of systemic lupus erythematosus

**Figure 1:**
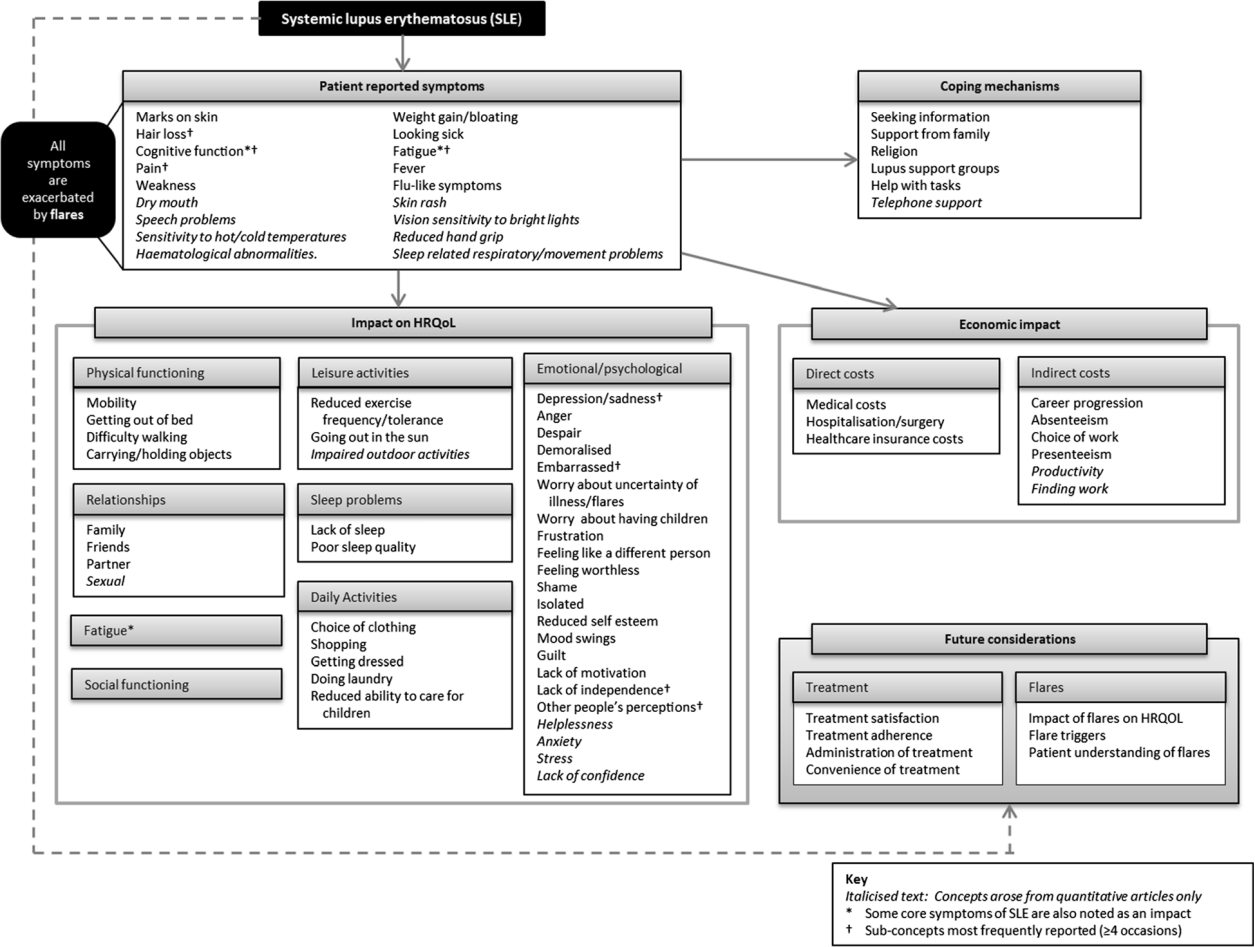
Systemic lupus erythematosus conceptual model.

Of the 38 articles that included patient-reported data, six were qualitative articles [22],[24]–[28] and 32 were quantitative articles. The resulting conceptual model shows the symptoms and impacts identified as key concepts related to SLE (Figure 1).

Fatigue and pain were identified as two of the most important and frequent symptoms for patients with SLE [24]–[26],[29]–[32] (Figure 1). Specifically, patients describe mental and physical symptoms of fatigue including impacts on social life [24], emotional wellbeing [24],[33], physical functioning [22],[24], sleep [31],[34]–[36] and the ability to complete daily tasks and leisure activities [27],[28]. Important cognitive symptoms include being “unable to think clearly” [22] and memory loss [22]. Other SLE symptoms include skin rash [27],[37], weight gain [24],[27] and hair loss [25],[27].

Symptoms impact all areas of HRQoL, with detrimental consequences observed in the physical, emotional and social functioning of SLE patients, as well as in their working life (Figure 1). In terms of the impact on emotional wellbeing, patients with SLE frequently feel sad, depressed, angry and demoralised [22],[24],[25],[30],[37],[38]. In particular, patients feel embarrassed [24],[25],[39] or self-conscious, or they lack self-esteem, primarily because of the change in their appearance (such as hair loss and skin manifestations) [22],[26]. Patients fear their disease worsening, and experience anxiety or stress related to the symptoms and the unpredictability of SLE [27],[30],[37],[38]. Many also experience feelings of frustration and a lack of 1) confidence, 2) independence, 3) control over one’s life and 4) belonging [40].

SLE has a significant negative impact on patients’ physical functioning, such as walking difficulty and other mobility problems [10],[22],[26],[41] (Figure 1). This affects various daily activities including opening jars and moving heavy objects [41], shopping [22], doing laundry [26], getting dressed [26] and caring for their children [24],[26]. Wider impacts on social functioning and working life are also reported [29],[40]. Specifically, patients have difficulty maintaining family and sexual relationships [24],[26],[37]. SLE also impacts negatively on patients’ career progression [25], absence from work [22], difficulty concentrating at work or study [22],[26],[32] and their choice of work [26],[27].

The conceptual model presented in this paper suggests that patients use various coping mechanisms for the unpredictability of flares, including 1) seeking and using information, 2) seeking emotional and practical help via the internet, 3) receiving support from hospital meetings, 4) receiving support from family, 5) attending lupus support groups and 6) religious practice [24],[26],[27] (Figure 1). The conceptual model also includes concepts such as treatment satisfaction, adherence and the impact of flares in a ‘future considerations’ box. There was a lack of evidence pertaining to these concepts in the currently available literature.

The conceptual model also demonstrates the economic burden of disease, in particular the high medical costs associated with SLE compared to other chronic diseases [42]. Substantial levels of inpatient care, medication/prescriptions and visits to healthcare professionals (HCP), which are all increased by ‘flares’, are the main drivers of direct costs in the treatment of SLE [43]. The conceptual model also shows that SLE is associated with high indirect costs due to lost productivity [5] resulting from unemployment and absenteeism, [44], with ‘in-flare’ patients with SLE having increased frequency and duration of time off work [45],[46].

A comparison between this conceptual model and the model developed by Gallop et al. [22] suggests similarities, although our model provides a broader perspective on the impact of SLE. There is alignment between the models on symptoms such as pain, fatigue, skin rash and hair loss. Impact on work/employment, impact on daily life/daily activities, lack of independence and emotional wellbeing are also presented in both models. In terms of differences, Gallop’s model [22] viewed impacts on social, family and leisure activities as one concept; however, the present literature review and model identified these as separate concepts. The emotional impact is conceptualised as depression or frustration in Gallop’s model [22], whilst a broader range of emotions associated with SLE (including worry, anger, embarrassment and shame in addition to depression and frustration) were identified in the literature. Unlike the model presented in this paper, impacts on physical functioning, relationships, sleep, worry about the ability to conceive/have children, the impact of others’ perceptions and the personal and broader economic burden are not shown in Gallop’s model. Whilst fatigue is noted as a symptom in both models, the model presented in this paper also conceptualises fatigue as an impact of SLE. Gallop et al. [22] include appearance and cognition concepts as impacts of SLE but these are captured as symptoms in the present model. Gallop et al. [22] also recognise the different triggers that may lead to the onset or worsening of symptoms in their conceptual model; however these issues were not identified in the present literature review.

### Review of patient-reported outcomes measures

A total of 23 PRO measures were identified from the articles reviewed. Of those, 15 measures targeted key concepts in the conceptual model and were therefore selected for in-depth review (Table 1). SLE-specific and generic PROs were categorised according to the concepts measured.

**Table 1:** Patient-reported outcome measures reviewed

**Instrument**	**Number of items**	**Recall period**	**Response format**	**Reference or rationale for inclusion of PRO if not cited in the literature search**
Systemic Lupus Activity Questionnaire (SLAQ) [47]	28	Last 3 months	First 27 items have 4-point scale, ranging from ‘none’ to ‘severe’. Last item has a 1–10 numerical rating scale (NRS)	[47]–[49]
LupusQoL [50]	34	Last 4 weeks	5-point scale ranging from ‘never’ to ‘all of the time’	[30],[51]
LupusPRO [52]	43	Last 4 weeks	5-point scale ranging from ‘never’ to ‘all of the time’	[52],[53]
L-QoL [40]	25	At the moment	Dichotomous ‘true’/’not true’ scale	[30],[40],[54]
SLEQOL [55]	40	Last week	All 7 point Likert scales, although scales have different labels (i.e. ‘not difficult at all’ to ‘very difficult’; ‘not at all’ to ‘extremely troubled’; ‘not at all’ to ‘extremely often’)	[30],[54]
Short Form 36 version 2 (SF-36v2) [56]	36	Standard 4-week recall or Acute 1-week recall version.	Varies dependent on scale [Yes/No and 3, 5 and 6-point Likert scales].	[30],[47],[49],[54],[57]–[63]
Euroqol 5 Domain (EQ-5D) (3-Level and 5-Level) [64],[65]	6	Recall of ‘*today*’ for both 3L and 5 L version.	3-point scale on 3L and 5-point scale on 5 L version, ranging from absence to inability to perform activity or extreme severity of symptom	Previously used in patients with SLE, and normative data exist for comparison. EQ-5D is a measure used to derive utilities
Functional Assessment of Chronic Illness Therapy-Fatigue Scale (FACIT-Fatigue) [66]	13	Past 7 days	5-point scale ranging from ‘not at all’ to ‘very much’.	[54]
Multidimensional Fatigue Inventory (MFI) [67]	20	Lately	5-level agreement scale from ‘Yes, that is true’ to ‘No, that is not true’.	[35]
Multidimensional Assessment of Fatigue (MAF) [68]	16	Past week	1-10 numerical rating scale (‘not at all’ – ‘a great deal) for items 1–14. Multiple-choice response for items 15 and 16.	[35]
McGill Pain Questionnaire (MPQ) [69]	20	Present pain	Mixed, ranging from 2-point to 6-point scales	[70]
Brief Pain Inventory – Short Form (BPI-SF) [71]	15	Last 24 hours	Most items have an 11 point numerical rating scale, ranging from 0 = no pain and 10 = pain. One item has a binary yes/no response and another ask patients to shade a diagram to show where they have pain. One item has a 0%-100% scale increasing in 10% increments.	Pain is a key symptom of SLE and the BPI-SF has previously been used in a SLE population
Beck Depression Inventory (BDI) [72]	21	Not specified	4-point scale with detailed wording provided for each response.	Depression is a key concept in SLE. The BDI has been used previously with patients with SLE, and normative data exist for comparison
Hospital Anxiety and Depression Scale (HADS) [73]	14	Past week	4-point scale	Depression is a key concept in SLE. The HADS has been used previously with patients with SLE and normative data exist for comparison
Work Productivity and Activity Impairment Questionnaire: Lupus (WPAI:Lupus) [74]	6	Past 7 days	Yes/No & NRS	Absenteeism and presenteeism is a major burden for patients with SLE. The WPAI:Lupus has been used previously in patients with SLE and normative data exist for comparison.

### Content validity – conceptual coverage

An overview of the conceptual coverage of each PRO measure was performed to evaluate content validity (Table 2). Of all 15 PROs reviewed, only the disease-specific *LupusQoL*, *LupusPRO, L-QoL* and *SLEQOL* have documented evidence for involvement of patients with SLE in concept elicitation and development of items [50],[52],[54],[55].

**Table 2:** **Content validity of patient-reported outcome measure**^**~**^

**Concept identified in literature review**	**SLE-specific HRQoL**					**Generic HRQoL**		**Fatigue**			**Pain**		**Depression**		**Work**
	**SLAQ**	**LupusQoL**^**$**^	**LupusPRO**^**$**^	**L-QoL**	**SLEQOL**	**SF-36v2**	**EQ-5D**^**#**^	**FACIT-Fatigue**	**MFI**	**MAF**	**MPQ**	**BPI-SF**	**BDI**	**HADS**	**WPAI: Lupus**
**Disease-defining signs and symptoms**															
Marks on skin	✓														
Weight gain/bloating	✓	✓													
Hair loss	✓	✓	✓												
Looking sick															
Reduced cognitive function	✓	✓	✓		✓				✓						
Fatigue	✓	✓	✓	✓	✓	✓		✓*	✓*	✓*			✓		
Pain	✓		✓		✓	✓	✓				✓	✓			
Fever	✓														
Weakness						✓		✓*	✓*						
Flu-like symptoms															
**Impacts**															
Physical functioning		✓	✓		✓	✓	✓		✓*	✓*		✓*			
Daily activities		✓	✓	✓	✓	✓	✓	✓*	✓*	✓*		✓*			✓
Sleep problems		✓				✓		✓*	✓* (p)			✓*			
Emotional/Psychological	✓	✓	✓	✓	✓	✓	✓	✓*		✓* (p)		✓*	✓	✓	
Social functioning		✓		✓	✓	✓		✓*	✓* (p)	✓*					
Leisure activities		✓			✓	✓			✓*	✓*					
Relationships		✓		✓		✓				✓ (p)		✓*			
**Work & economic impacts**															
Work disability			✓		✓	✓				✓*		✓*			✓
Economic impact			✓		✓	✓									
**Total**	**8/27**	**11/27**	**9/27**	**5/27**	**10/27**	**12/27**	**4/27**	**6/27**	**8/27**	**8/27**	**1/27**	**7/27**	**2/27**	**1/27**	**2/27**

SLAQ – Systemic Lupus Activity Questionnaire, SF-36v2 – Short Form [36] Health Survey, EQ-5D – EuroQol 5-Dimensions, FACIT-Fatigue - Functional Assessment of Chronic Illness Therapy, MFI – Multidimensional Fatigue Inventory, MAF – Multidimensional Assessment of Fatigue, MGQ – McGill Pain Scale, BPI – Brief Pain Inventory, BDI – Beck Depression Inventory, HADS – Hospital Anxiety and Depression Scale, WPAI:Lupus – Work Productivity and Activity Impairment Questionnaire: Lupus.

^~^ The following symptoms were identified as important in the literature review, but are not measured by current PROs: flu-like symptoms, dry mouth, skin rash, speech problems, vision sensitivity to bright lights, sensitivity to hot/cold temperatures, reduced hand grip, haematological abnormalities, sleep related respiratory and movement problems ^$^PRO measure has documented evidence of qualitative research with patients with SLE during item generation ^#^Result valid for both the EQ-5D-3L and EQ-5D-5L *Concept assessed only as a fatigue related impact (p) Concept partially covered by one or more items.

In terms of the SLE-specific and generic HRQoL instruments, the *SF-36v2*, *LupusQoL* and *SLEQOL* demonstrated a greater level of conceptual coverage, in terms of the variety and number of relevant SLE concepts measured, compared to the other four HRQoL instruments (Table 2).

All three fatigue PROs demonstrated similar levels of conceptual coverage for SLE, with each measuring fatigue and its impact on daily activities and social functioning (Table 2). The *MAF* was the only fatigue-related PRO to measure the impact of fatigue on relationships (i.e. sexual activity or socialising with friends) and ability to work. Of the two pain-related PROs, the *BPI-SF* had superior conceptual coverage compared to the *MPQ*, measuring both pain symptoms and the impact of pain on a range of aspects of daily life.

As expected, both depression-related PROs were focussed on the emotional/psychological well-being impact, but the *BDI* also measures fatigue (‘tiredness’), patients’ attitudes towards their appearance and impact on sleep and ability to work – concepts which are also relevant to SLE patients (Table 2). The *WPAI:Lupus* measures relevant SLE concepts such as ability to work, disability associated with lupus and the impact of lupus on activities of daily living such as housework and childcare.

### Face validity

Of all the PRO measures reviewed, only the *LupusQoL, LupusPRO and L-QoL* had documented evidence of qualitative involvement of patients with SLE to evaluate the face and content validity through cognitive debriefing techniques (Table 3). The *LupusPRO* was pilot tested with SLE patients, though the face validity was not qualitatively assessed. Patient insights were used in the development of the *SLEQOL*, although only by means of ranking items in order of importance; the face validity was not qualitatively assessed. A face validity review suggested that the instructions and item wording, recall period and response options for six PRO measures (*L-QoL, SF-36v2, EQ-5D-3L, EQ-5D-5L, FACIT-Fatigue, HADS*) appeared acceptable and appropriate for a SLE population and are in line with guidance for the use of PROs in clinical trials [11] (Table 3). The standard version of the *SF-36v2* has a four week recall period, which is unlikely to be acceptable to regulators. However, the acute version, with a seven day recall period, is likely to be more acceptable. The item and instruction wording of each PRO measure was clear, free of clinical terminology and generally written in simple language. The response options of these six PROs are generally clear and appropriate for patients with SLE. Furthermore, the recall periods were appropriate for intended use of the measures with patients with SLE.

**Table 3:** Content and face validity of each patient-reported outcome measure

**PRO Instrument**	**Content and face validity evaluated with SLE patients using cognitive debriefing methodology**	**Item wording acceptable**	**Recall period acceptable for patient population**	**Response options acceptable**
**SLE-specific HRQoL**				
SLAQ	✗	✓	✗	✓
LupusQoL	✓	✓	✗	✓
LupusPRO	✓	✓	✗	✓
L-QoL	✓	✓	✓	✓
SLEQOL	✗	✗ ‘Fatigue’ not defined	✓	✗ Confusing
**Generic HRQoL**				
SF-36v2	✗	✓	✗	✓
EQ-5D^#^	✗	✓	✓	✓
**Fatigue**				
FACIT-Fatigue	✗	✓	✓	✓
MFI	✗	✓	✗ ‘Lately’ too vague	✗ Confusing
MAF	✗	✗ ‘Fatigue’ not defined	✓	✗ Confusing
**Pain**				
MPQ	✗	✗ Complex terms used	✓	✗ Complex terms used
BPI-SF	✗	✓	✗ Some items don’t specify recall	✓
**Depression**				
BDI	✗	✗ No question wording	✗ None specified	✗ Lengthily
HADS	✗	✓	✓	✓
**Work productivity**				
WPAI:Lupus	✗	✗ Some complex terms used	✓	✓

SLAQ – Systemic Lupus Activity Questionnaire, SF-36v2 – Short Form [36] Health Survey, EQ-5D – EuroQol 5-Dimensions, FACIT-Fatigue - Functional Assessment of Chronic Illness Therapy, MFI – Multidimensional Fatigue Inventory, MAF – Multidimensional Assessment of Fatigue, MGQ – McGill Pain Scale, BPI – Brief Pain Inventory, BDI – Beck Depression Inventory, HADS – Hospital Anxiety and Depression Scale, WPAI:Lupus – Work Productivity and Activity Impairment Questionnaire: Lupus. ^#^Result valid for both the EQ-5D-3L and EQ-5D-5L.

In contrast, the other nine PROs included features which may hinder understanding and completion of the items by patients (Table 3). Four PROs used vague and insufficiently defined (e.g. ‘leisure and recreational activities’ and ‘fatigue’ in the *MAF*) or complex terminology (e.g. ‘lacerating’ and “spatial” in the *MPQ*) in the items and instructions. The individual instructions provided for the *WPAI:Lupus* items increase the word count/length, which may be overwhelming for some patients.

The format of the response options for some PROs was inconsistent, which may cause confusion to patients (*SLEQOL*, *MFI, MAF*); some used complex terms such as ‘excruciating’ (*MPQ*) or were lengthy and ambiguous (*BDI*) (Table 3).

### Psychometric properties

The *LupusQoL, EQ-5D* and the *FACIT-Fatigue* demonstrated the strongest psychometric properties in an SLE population [47],[50],[75]–[77] (Table 4). Psychometric validation of the *SLAQ*, *LupusPRO* and *SF-36v2* has also been conducted in SLE [47],[52],[76],[78],[79]. None of the other nine PROs had documented evidence of their measurement properties in patients with SLE. It is important to acknowledge that many recent randomised controlled trials (RCTs) in SLE have not incorporated the use of a patient global assessment of disease activity, thus making it difficult to determine minimal important differences (MIDs) for PRO measures. This may contribute to the lack of evidence pertaining to MIDs in SLE.

**Table 4:** Psychometric properties of patient-reported outcome measures in patients with SLE

**Instrument**	**Psychometrically validated in a SLE population**	**Reliability**		**Validity**			**Ability to detect change**
		**Internal consistency (Cronbach’s α)**	**Test-retest (ICC)**	**Construct validity**	**Known groups validity**	**Concurrent validity**	
SLAQ	✓ [47],[78]	✓ (0.87) [47]	-	✓ [47],[50]	-	✓ [78]	✓ [47]
LupusQoL	✓ [50]	✓ (>0.70) [50]	✓ (0.72-0.93) [50]	✓ [50]	✓ [50]	✓ [50]	-
LupusPRO	✓ [52],[76]	✓ (0.72-0.94) [52]	✓ (>0.70, except for 3 domains) [52]	✗ (High floor effects) [52] -	✓ [52],[76]	✓ [52]	-
L-QoL	✓ [40]	✓ (0.92) [40]	✓ (0.92) [40]	-	✓ [40]	✓ [40]	-
SLEQOL	✓ [55]	✓ (0.95) [55]	✓ (0.83) [55]	✗ (High floor effects) [55]	-	Partial [55]	Partial [55]
SF-36v2	✗	*Partial* (>0.70) [80],[81]	*Partial*[80],[81]	*Partial*[80],[81]	*Partial*[80],[81]	*Partial*[80],[81].	✗ (for SLE) [55],[60]*Partial*[80]
EQ-5D	✓ [75]	-	EQ-5D Index: *Partial* (0.89) [75] EQ-5D VAS: *Partial* (0.77) [75]	✗ [82]	✓ [76]	✓ [76]	✓ [76]
FACIT-Fatigue	✓ [77]	✓ (>0.95) [77]	*Partial* (0.95) [83]	-	✓ [77]	✓ [77]	✓ [77]
MFI	✗	*Partial* (0-68-0.89) [84]	-	*Partial*[84],[85] I	*Partial*[84],[86]	*Partial*[84]	*Partial*[87]
MAF	✗	*Partial* (0.93) [68]	*Partial* (0.87) [88]	*Partial*[89]	*Partial*[90]	*Partial*[68]	*Partial*[91]
MPQ	✗	*Partial* (α not provided) [69]	*Partial* (r= > 0.70) [69],[92]	-	-	*Partial*[93]	*Partial*[94]
BPI-SF	✗	*Partial* (0.80-0.92) [95]	*Partial* (0.83-0.88) [96]	*Partial*[95],[97],[98]	*Partial*[95],[99]	*Partial*[99]	*Partial*[100],[101]
BDI	✗	*Partial* (0.81) [102]	*Partial* (0.93) [102]	*Partial*[102],[103]	*Partial*[102],[103]	*Partial*[102]	-
HADS	✗	*Partial* (0.83, Anxiety sub-scale; 0.82, Depression sub-scale) [104]	*Partial* (0.84-0.85 for both sub-scales) [105]	*Partial*[73],[104]	*Partial*[105]	*Partial*[104],[106]	*Partial*[105]
WPAI:Lupus	✗	-	*Partial* (Pearson’s: 0.71-0.75) [74]	-	*Partial*[107]	*Partial*[108]	*Partial*[107]

SLAQ – Systemic Lupus Activity Questionnaire, SF-36v2 – Short Form [36] Health Survey, EQ-5D – EuroQol 5-Dimensions, FACIT-Fatigue - Functional Assessment of Chronic Illness Therapy, MFI – Multidimensional Fatigue Inventory, MAF – Multidimensional Assessment of Fatigue, MGQ – McGill Pain Scale, BPI – Brief Pain Inventory, BDI – Beck Depression Inventory, HADS – Hospital Anxiety and Depression Scale, WPAI:Lupus – Work Productivity and Activity Impairment Questionnaire: Lupus.

✓, evidence of psychometric acceptability in SLE population; *Partial*, evidence of psychometric acceptability in non-SLE population; ✗, evidence does not meet psychometrical acceptability threshold; −, no evidence available.

#### Systemic lupus erythematosus specific health-related quality of life measures

All five SLE-specific HRQOL measures reviewed in depth had sufficient evidence of internal consistency (i.e. α > 0.7) [40],[47],[50],[52],[55] (Table 4). The *LupusQoL, L-QoL* and SLEQOL had evidence of strong test–retest reliability (i.e. ICC > 0.7) [40],[50], while the test–retest was shown for *LupusPRO* for only 7 out of its 11 domains [52]. The *SLAQ* had no evidence of test–retest reliability. Only the *LupusQoL* and *L-QoL* had sufficient evidence of validity for a SLE population [50]. While the *SLAQ* demonstrated construct validity [47], it had no evidence that it is capable of differentiating known groups, and the *LupusPRO* demonstrated high floor effects [52]. The *LupusQoL* and *LupusPRO* had no evidence of ability to detect change, while the *SLAQ* demonstrate a limited sensitivity to a change in health status but no evidence of responsiveness to changes to treatment [47].

#### Generic health-related quality of life measures

[79] Both of the generic HRQoL measures reviewed had sufficient evidence of test–retest reliability [75],[80],[81] and both demonstrated sufficient evidence of validity [80],[81] (Table 4).

The *SF-36v2* has been validated in many different health conditions and is a widely used and accepted measure of HRQoL [80],[81]. More importantly, in an SLE population, the *SF-36v2* has demonstrated evidence of internal consistency reliability, concurrent validity and known groups validity [79]. The *EQ-5D* showed evidence of validity and an ability to detect change in patients with SLE [76] despite being commonly associated with ceiling and floor effects [82]. Of note, the *SF36v2* is able to detect change in many conditions, including rheumatoid arthritis [80],[81] and more recently, distribution and anchor-based estimates suggest MIDs of approximately 3–6 points in an SLE population [79].

#### Fatigue measures

Of the fatigue measures, the *FACIT-Fatigue* demonstrated the strongest evidence of internal consistency reliability, known groups validity, concurrent validity, ability to detect change in a SLE population [77] (Table 4) and test-retest reliability in psoriatic arthritis [83]. Furthermore, the *FACIT-Fatigue* has shown strong evidence of internal consistency reliability and known groups validity, with an MID of 3–4 points in rheumatoid arthritis [91]. The *MFI* and *MAF* had sufficient evidence of internal consistency [68],[84], though Cronbach’s α for one of the *MFI* domains was below 0.7 [84], and the *MAF* had evidence of test–retest reliability (in cancer) [88]. While not validated in a SLE population, the *MFI* and *MAF* had sufficient evidence of validity and an ability to detect for Sjögren’s syndrome and rheumatoid arthritis (other autoimmune inflammatory conditions) [68],[84],[86],[89],[90] but evidence of the ability of the *MAF* to detect change was limited to cancer patients [87].

#### Pain measures

In terms of the pain items, neither the *BPI-SF* nor *MPQ* have been validated in SLE (Table 4). In other conditions, the *BPI-SF* demonstrated the strongest evidence of both internal consistency (α > 0.7) and test-retest reliability (ICC > 0.7) [95],[96]. The *MPQ* had sufficient evidence of test-retest reliability also [69],[92] but only provided partial evidence of internal consistency [69]. The *BPI-SF* had acceptable evidence of validity [95],[97]–[99]. In contrast, the *MPQ* had evidence of concurrent validity only [93]. The *MPQ* and *BPI-SF* demonstrated an ability to detect changes, with both providing evidence of responsiveness to treatment in musculoskeletal pain (*MPQ*) [94], cancer and rheumatoid arthritis patients (*BPI-SF*) [100],[101].

#### Depression measures

Neither the *BDI* nor *HADS* have been validated in SLE. However, both measures have evidence of reliability in other conditions [102],[104],[105] (Table 4). Both measures have sufficient evidence of validity in the general population and psychiatric patients [73],[102]–[106], though the construct validity of the *BDI* varies, with the number of factors ranging from three to seven depending on the disease [102]. Of the two depression measures, only the *HADS* provides evidence of ability to detect change in response to intervention in different diseases, including depression, neurotic disorder, cancer and heart disease [105].

#### Work productivity measure

There is no documented evidence of the psychometric properties of the *WPAI:Lupus* in SLE, though item content in the general health version (*WPAI:GH)* is highly consistent with the ‘specific health problem’ version of the measure (*WPAI:SHP*) [109]. The content and item wording of the *WPAI:GH* and the *WPAI:SHP* is the same, with the exception of the term ‘general health’ in the *WPAI:GH*, which is replaced with the relevant disease. For the *WPAI:Lupus,* ‘general health’ is replaced with ‘lupus’. Therefore, the validity of the *WPAI:Lupus* can be partially demonstrated by acceptable concurrent and known groups validity of the general health version of the measure (*WPAI:GH*) in the general population [74] and in rheumatoid arthritis [108]. The *WPAI:GH* also had evidence of test–retest reliability in rheumatoid arthritis [74] but none of internal consistency. Moreover, the ankylosing spondylitis-specific *WPAI:SpA* has shown known groups validity and responsiveness to treatment of ankylosing spondylitis [107].

## Discussion

To understand the value of therapies for SLE from the patient-perspective, PRO measures should be included in clinical trials in conjunction with well-established clinical assessments. The selection of suitable measures to assess SLE-related symptoms and impacts in clinical trials requires careful consideration [14],[15]. This study therefore sought to develop a conceptual model of the key symptoms and impacts associated with SLE to help support identification of suitable endpoints for clinical trials in SLE [14],[15]. The model also aimed to integrate the economic burden of SLE to patients, health care providers and the wider society.

The key patient-reported concepts identified within the model were fatigue, pain, cognition, daily activities, emotional well-being, physical/social functioning and work productivity. The subjective nature of many SLE symptoms and impacts requires accurate and reliable measurement of these symptoms based on patient self-report. In light of this, our study also sought to review and evaluate the face and content validity and psychometric properties of PROs that may be appropriate for use in a SLE population. To our knowledge, this is the first comprehensive review of PROs for the whole range of key symptoms and impacts experienced specifically by patients with SLE. The American College of Rheumatology conducted a review to recommend measures for inclusion in SLE trials [34], but this was limited to the evaluation of fatigue. In a review of outcome measures in SLE clinical trials, Strand et al. [54] included a brief summary of the measurement properties of a small number of selected HRQoL measures used previously in SLE trials, but this review did not include evaluation of face and content validity.

The current review showed that the *LupusQoL, SLEQOL*, *SF-36v2*, *FACIT-Fatigue and BPI-SF* demonstrated face and content validity and were psychometrically strong as measures of the key concepts identified in the conceptual model. The *FACIT-Fatigue* and *BPI-SF* appeared to be the strongest instruments. In addition, the generic *SF-36v2* and *EQ-5D* measures are widely used in trials with patients with SLE and are recognised and accepted by clinical, patient, regulatory, reimbursement and academic communities.

A recent qualitative study involving SLE patients concluded that the *FACIT-Fatigue* is a relevant measure of fatigue in SLE [28]. Furthermore, the psychometric properties of the *FACIT-Fatigue* in an SLE population are well documented [77]. In contrast, the American College of Rheumatology study from 2007 suggest the *Fatigue Severity Scale (FSS)* as a measure of fatigue in SLE [34]. However, our review questions the lack of SLE patient involvement in the initial development of the *FSS*; and the lack of supporting evidence of its face and content validity. Furthermore, the psychometric properties of the *FSS* have been assessed but in a limited number of patients with SLE [110],[111]. Indeed, of all the PRO measures reviewed, only three Lupus-specific measures (*LupusQoL, LupusPRO and L-QoL)* have documented evidence of qualitative input from patients with SLE.

Important in the measurement of SLE is to acknowledge that patients may experience many symptom-free days, followed by a severe flare. Flares are likely to impact patients’ health-related quality of life [7],[10], as well as having a wider society and economic impact [45]. For example, patients with flares incur higher direct and indirect costs compared with those without flares [45]. Therefore, PRO measurement of the impact of flares may be considered in future clinical trials in patients with SLE in addition to clinical outcome assessments [14],[15]. Despite this, no PRO measures were identified which target the impact of flares on humanistic burden. In addition, SLE often involves day-to-day symptom fluctuations due to these flares, thus the recall period of the measurement instrument is also an important consideration. PROs with shorter recall periods may underestimate symptom burden and may place undue demand on patients; however, longer recall period may not allow for reliable symptom and impact reporting.

Whilst the validity of this literature review is strengthened by the inclusion of qualitative and quantitative studies, with the review of PRO measures conducted in accordance with regulatory guidelines [11],[12],[23], there are inherent limitations. Firstly, an assessment of the quality of the studies identified from the literature search was not conducted, so as not to limit our search. The review was however intended to be as inclusive and wide-ranging as possible to capture all of the key concepts for the development of the conceptual model and to ensure that atypical symptoms were not missed. Secondly, after key SLE symptoms and impacts in the conceptual model were identified, PROs were selected for in-depth review on the basis of measurement of those fundamental symptoms and impacts. PRO measures of other symptoms of SLE not reported in the conceptual model were thus de-prioritised and therefore not included in the in-depth review. Nevertheless, PRO measures for some key concepts identified in the model (for example, skin manifestations of the disease, impact of flares and treatment satisfaction) were either not identified from the literature search, or no PRO has been used to measure these concepts in SLE. This represents a gap in knowledge which may benefit from further research.

PROs are acknowledged as complementary to more objective measures and are being incorporated more frequently into clinical trials and clinical practice [112]. The recognition and measurement of disease experience from the patient’s perspective is an important factor for SLE clinical trial design and health care decision makers. The results of the present study, demonstrating the suitability of PROs for use in clinical trials of SLE within the guidance provided by the FDA and EMA [11],[12],[14],[15], can hopefully be of benefit to clinical research within SLE.

## Conclusion

SLE is a condition associated with high unmet need and considerable burden to patients, as demonstrated by the conceptual model presented in this paper. This review highlights the existing patient-reported measures of HRQoL, SLE signs and symptoms and work productivity that demonstrate appropriate content and face validity and are psychometrically adequate for a population of patients with SLE, and as a result such measures may be suitable for use in SLE clinical trials.

## Abbreviations

BDI: Beck depression inventory

BPI-SF: Brief Pain Inventory – Short Form

EMA: European Medicines Agency

FDA: Food and Drug Administration

HRQoL: Health-related quality of life

IQWIG: Germany, Institute for Quality and Efficiency in Healthcare

LN: Lupus Nephritis

MPQ: McGill Pain Questionnaire

MAF: Multidimensional Assessment of Fatigue

NICE: UK, National Institute of Health and Clinical Excellence

PROs: Patient-reported Outcomes

SLE: Systemic lupus erythematosus

FACIT-Fatigue: The Functional Assessment of Chronic Illness Therapy - Fatigue Scale

HADS: The Hospital Anxiety and Depression Scale

WPAI:Lupus: Work Productivity and Activity Impairment Questionnaire: Lupus

## Competing interests

The study was funded by Novo Nordisk A/S, Denmark and conducted by Adelphi Values. LHo, LHu, LHe, CP and HK are employees of Adelphi Values (or were employees at the time the research was conducted). LHø, MS-L, and BBH are employees of Novo Nordisk A/S.

## Authors’ contributions

LHo, LHu, LHe, CP and HK participated in the study conception and design, analysis and interpretation of literature, development of the conceptual model, and reviewed and approved the manuscript. LHø, MS-L, and BBH participated in the study conception and design, analysis and interpretation, reviewed and approved the final version of the manuscript. All authors read and approved the final manuscript.
